# Applicability of the WHO maternal near-miss tool: A nationwide surveillance study in Suriname

**DOI:** 10.7189/jogh.10.020429

**Published:** 2020-12

**Authors:** Kim JC Verschueren, Lachmi R Kodan, Raëz R Paidin, Sarah M Samijadi, Rubinah R Paidin, Marcus J Rijken, Joyce L Browne, Kitty WM Bloemenkamp

**Affiliations:** 1 Department of Obstetrics, Division Women and Baby, Birth Centre Wilhelmina’s Children Hospital, University Medical Center Utrecht, Utrecht University, Utrecht, the Netherlands; 2 Department of Obstetrics, Academic Hospital Paramaribo, Paramaribo, Suriname; 3 Department of Obstetrics, Diakonessen Hospital Paramaribo, Paramaribo, Suriname; 4 Julius Global Health, Julius Centre for Health Sciences and Primary Care, University Medical Centre Utrecht, Utrecht University, Utrecht, the Netherlands

## Abstract

**Background:**

Maternal near-miss (MNM) is an important maternal health quality-of-care indicator. To facilitate comparison between countries, the World Health Organization (WHO) developed the “MNM-tool”. However, several low- and middle-income countries have proposed adaptations to prevent underreporting, ie, Namibian and Sub-Sahara African (SSA)-criteria. This study aims to assess MNM and associated factors in middle-income country Suriname by applying the three different MNM tools.

**Methods:**

A nationwide prospective population-based cohort study was conducted using the Suriname Obstetric Surveillance System (SurOSS). We included women with MNM-criteria defined by WHO-, Namibian- and SSA-tools during one year (March 2017-February 2018) and used hospital births (86% of total) as a reference group.

**Results:**

There were 9114 hospital live births in Suriname in the one-year study period. SurOSS identified 71 women with WHO-MNM (8/1000 live births, mortality-index 12%), 118 with Namibian-MNM (13/1000 live births, mortality-index 8%), and 242 with SSA-MNM (27/1000 live births, mortality-index 4%). Namibian- and SSA-tools identified all women with WHO-criteria. Blood transfusion thresholds and eclampsia explained the majority of differences in MNM prevalence. Eclampsia was not considered a WHO-MNM in 80% (n = 35/44) of cases. Nevertheless, mortality-index for MNM with hypertensive disorders was 17% and the most frequent underlying cause of maternal deaths (n = 4/10, 40%) and MNM (n = 24/71, 34%). Women of advanced age and maroon ethnicity had twice the odds of WHO-MNM (respectively adjusted odds ratio (aOR) = 2.6, 95% confidence interval (CI) = 1.4-4.8 and aOR = 2.0, 95% CI = 1.2-3.6). The stillbirths rate among women with WHO-MNM was 193/1000births, with six times higher odds than women without MNM (aOR = 6.8, 95%CI = 3.0-15.8). While the prevalence and mortality-index differ between the three MNM tools, the underlying causes of and factors associated with MNM were comparable.

**Conclusions:**

The MNM ratio in Suriname is comparable to other countries in the region. The WHO-tool underestimates the prevalence of MNM (high mortality-index), while the adapted tools may overestimate MNM and compromise global comparability. Contextualized MNM-criteria per obstetric transition stage may improve comparability and reduce underreporting. While MNM studies facilitate international comparison, audit will remain necessary to identify shortfalls in quality-of-care and improve maternal outcomes.

Sustainable Development Goal target 3.1 aims to eliminate preventable maternal deaths and reduce the global maternal mortality ratio (MMR) to less than 70 per 100 000 live births (LB) by 2030 [1]. Women who die represent just the tip of the iceberg: for each woman who dies, at least ten suffer from severe maternal complications and narrowly escape death by chance or because of the care they receive: a maternal near-miss (MNM) [2]. With the decline of maternal deaths, MNM is used as a proxy to measure the quality of obstetric care [2,3]. MNM has the advantage that it occurs more frequently and that the survival of the woman makes it less threatening to report by health care providers [2-4]. In Suriname, a middle-income country in South America, the MMR is 130 per 100 000 LB, one of the highest in the Caribbean & America’s, but the absolute number of deaths is “only” ten to fifteen per year [5]. This makes MNM studies crucial to develop justified recommendations and finally reduce maternal mortality [2,4].

To standardize the MNM definition and facilitate comparison between different countries, the World Health Organization (WHO) developed the “Maternal near-miss approach” in 2011 [2]. The classification includes three types of criteria: disease-, intervention,- and organ dysfunction-based. If any organ dysfunction criteria are met, the MNM approach defines the case as ‘life-threatening’ and therefore, MNM. The choice for organ-dysfunction criteria follows the concept that the following sequence of events leads from good health to death: clinical disease, systemic inflammatory response syndrome, organ dysfunction, organ failure and finally death [6,7]. Following this concept, organ dysfunction markers (25 criteria) define MNM [2]. However, several studies in different settings demonstrated that the organ-dysfunction criteria may not be suitable and proposed adapted criteria to prevent underreporting of life-threatening disorders [8-12]. In 2017, a Delphi study suggested adaptations to the WHO-criteria for low-resource settings in Sub-Sahara Africa (SSA) [10]. The adapted MNM tool included several clinical conditions, such as eclampsia, sepsis and uterine ruptured and a lower threshold for blood transfusion, and performed well in Ethiopia [11]. A recent study in Namibia suggested that both tools were not suitable for middle-income countries and proposed criteria ‘in-between’ WHO-MNM and SSA-MNM [12]. However, the resulting heterogeneity of these adapted MNM criteria compromises comparability [3], which the WHO approach specifically intended to avoid.

The goal of studying maternal near-miss in Suriname would be to (1) find a reason for the relatively high maternal mortality, and stillbirth rate in the country [5,13,14], (2) compare findings to other countries and (3) improve the quality of care. Due to the variety of (adapted) MNM-criteria, it is unclear which criteria are most applicable to achieve the abovementioned aims. Therefore, this nationwide study in Suriname first aims to apply the WHO-MNM tool and adapted Namibian and SSA-tools to evaluate differences in prevalence, mortality-index, underlying causes, and factors associated with maternal near-miss. The comparison of MNM in a clinical setting may facilitate possible amendments of the global WHO near-miss criteria to assure uniformity and applicability.

## METHODS

### Study design and setting

A prospective nationwide population-based cohort study, using the Suriname Obstetric Surveillance System (SurOSS), was performed during one year (March 2017 to February 2018). Suriname is situated on the Northern coast of South-America, with a population of approximately 560 000 and 10 000 live births a year [15]. The five hospitals conduct approximately 86% of all births, 4% women deliver at home, 6% of women deliver at the primary health care services and in 4% the place of birth is unknown [15]. In general, all women with (severe) morbidity are referred to a hospital. Maternal deaths (in facilities and the community) are reported to the Surinamese Maternal Mortality Committee. For a detailed description of the health care system, see our previous publications on maternal mortality and childbirth outcomes [5,13,14,16].

### Maternal near-miss case definition

Within SurOSS we identified all women with potentially-life threatening complications (PLTC, ie, disease- and intervention-criteria) and life-treatening complications (LTC, ie, MNM, organ dysfunction criteria) according to the WHO near-miss approach [2]. Per Surinamese Maternal Mortality Committee consensus directions, the criteria were minorly, contexually adaptated to clarify definitions and prevent inclusion of women without PLTC (**Table 1**), as follows:

**Table 1:** Definition of potentially life-threatening and life-threatening complications in pregnancy defined by WHO and minor adaptations within the Suriname Obstetric Surveillance System (SurOSS)

Criterion	WHO	SurOSS
**Potentially life-threatening complications (PLTC)**		
**Disease-based criteria:**		
**Severe post-partum hemorrhage**	Genital bleeding after delivery, with at least one of the following: perceived abnormal bleeding (1000 mL or more) or any bleeding with hypotension or blood transfusion.	• 1000 mL blood loss and/or
		• Any bleeding (antepartum, intrapartum or postpartum) with hypotension or transfusion of at least 3 products
**Severe pre-eclampsia**	Persistent systolic blood pressure of 160 mm Hg or more or a diastolic blood pressure of 110 mm Hg; proteinuria of 5 g or more in 24 h; oliguria of <400 mL in 24 h; and HELLP syndrome or pulmonary oedema. Excludes eclampsia.	Systolic blood pressure of 160 mm Hg or more, or diastolic blood pressure of 110 mm Hg or more on two occasions at least 4 h apart and:
		• Thrombocytopenia (platelet count of <100x9 10^9^/L)
		• Raised plasma ALT or AST (twice the upper limit of normal)
		• Renal insufficiency (doubling of the serum creatinine)
		• Pulmonary edema
		• Pre-eclampsia complaints, not attributed to other causes, such as unresponsive headache, epigastric pain, visual disturbances
**Eclampsia**	Generalized fits in a patient without previous history of epilepsy. Includes coma in pre-eclampsia.	Seizures in a woman during pregnancy or up to 14 d postpartum, without any other attributable cause, with at least one of the following signs:
		• Hypertension (≥140 mm Hg systolic or ≥90 mm Hg diastolic)
		• Proteinuria [at least 1 g/L [‘2 +’] on dipstick testing]
		• Thrombocytopenia (platelet count of <100x9 10^9^/L)
		• Raised plasma ALT or AST (twice the upper limit of normal)
**Severe sepsis**	Presence of fever (body temperature >38°C), a confirmed or suspected infection (eg, chorioamnionitis, septic abortion, endometritis, pneumonia), and at least one of the following: heart rate >90, respiratory rate >20, leukopenia (white blood cells <4000), leukocytosis (white blood cells >12 000).	Any pregnant or recently pregnant woman (up to 6 weeks postpartum) diagnosed with severe sepsis (irrespective of the source of infection). Clinical diagnosis of severe sepsis, associated with two or more of the following:
		• Temperature >38C or <36C measured on two occasions at least 4 h apart
		• Heart rate >100 beats/min measured on two occasions at least 4 h apart
		• Respiratory rate >20/min measured on two occasions at least 4 h apart
		• White cell count >17x10^9^/L or <4x10^9^/L or with
		•>10% immature band forms, measured on 2 occasions
**Ruptured uterus**	Rupture of uterus during labour confirmed by laparotomy.	A visually confirmed, complete rupture of the myometrium and serosa
**Severe complications of abortion**	Not further defined	Severe hemorrhage (≥1000mL, hypotension, blood transfusion of at least 3 products), severe sepsis or complications due lesion of intestines or other organs or complications related to anesthesia.
**Intervention-criteria:**		
**Intensive care unit admission**	Not further defined	Admission to a ward where mechanical ventilation and administration of continous vasoactive drugs are possible
**Intervention radiology**	Not further defined	Not available in Suriname
**Laparotomy excluding caesarean section**	Not further defined	Excluding uncomplicated laparotomy for ectopic pregnancy when patient remains hemodynamically stable and blood loss is less than 1000 mL and less than three blood products
**Use of blood products**	Not further defined	Use of at least 3 blood products
		Excluding blood transfusion for anaemia without any other complications
**Life-threatening**		
**Organ-dysfunction criteria:**		
**Cardiovascular**	Shock, cardiac arrest (absence of pulse/ heart beat and loss of consciousness), use of continuous vasoactive drugs, cardiopulmonary resuscitation, severe hypoperfusion (lactate >5 mmol/L or >45 mg/dL), severe acidosis (pH<7.1)	
**Respiratory**	Acute cyanosis, gasping, severe tachypnea (respiratory rate >40 breaths per minute), severe bradypnea (respiratory rate <6 breaths per minute), intubation and ventilation not related to anesthesia, severe hypoxemia (O2 saturation <90% for ≥60 min or PAO2/FiO_2_ < 200)	
**Renal**	Oliguria non-responsive to fluids or diuretics, dialysis for acute renal failure, severe acute azotemia (creatinine ≥300 μmol/mL or ≥3.5 mg/dL)	
**Coagulation / hematological**	Failure to form clots, massive transfusion of blood or red cells (≥5 units), severe acute thrombocytopenia (<50 000 platelets/mL)	
**Hepatic**	Jaundice in the presence of pre-eclampsia, severe acute hyperbilirubinemia (bilirubin >100 μmol/L or >6.0 mg/dL)	
**Neurologic**	Prolonged unconsciousness (lasting ≥12 h)/coma (including metabolic coma), stroke, uncontrollable fits/status epilepticus, total paralysis	
**Uterine dysfunction**	Uterine hemorrhage or infection leading to hysterectomy

1. Transfusion of one blood product was increased to ≥ three blood products and women were excluded who were transfused for only anaemia without any other complications;
2. Laparotomy for ectopic pregnancy was only included if blood loss was ≥1000 mL, blood was transfused or if patient was hemodynamically unstable [12];
3. Definition of maternal sepsis and eclampsia were harmonized with the United Kingdom (UKOSS) and International Network of Obstetric Surveillance System (INOSS) [17,18].

### Data collection

Eligible women were identified by the research coordinator (doctor) of each hospital during daily rounds. The authors weekly screened the medical files of all discharged women on the gynaecology and obstetric wards, in the intensive care of all hospitals. Additionally, the hospital registries reported whether patients on non-obstetric departments were consulted by a gynaecologist or obstetrician or had a ICD-code related to pregnancy. The research coordinator of the primary health care centers were contacted every quartile and reported women who were not transferred to a hospital.

Medical files were retrieved of all discharged women with PLTC and digitalized using an anonymous 188-item digital case report form on a password-secured Kobotoolbox. Data on demographics, general and obstetric history, occurrence of maternal and perinatal adverse outcomes were retrieved. The Surinamese Maternal Mortality Committee conducted verbal autopsy and audits of all maternal deaths and shared the elaborate case summaries.

For the purpose of this study, all maternal deaths in the study period and women with any WHO-MNM, Namibian-MNM or SSA-MNM were extracted for analysis (**Table 2**). The SSA-MNM criteria were developed after our study commenced [10]. This resulted in women who received two units of red blood cells without any other MNM-criteria not being included.

**Table 2:** MNM criteria according to the WHO, Namibian and Sub-Sahara Africa tools

	WHO	Namibian	SSA		WHO	Namibian	SSA
**Clinical criteria**				**Cardiovascular dysfunction**			
Acute cyanosis	**Yes**	**Yes**	**Yes**	Shock	**Yes**	**Yes**	**Yes**
Gasping	**Yes**	**Yes**	**Yes**	Cardiac Arrest	**Yes**	**Yes**	**Yes**
Respiratory rate >40 or <6/min	**Yes**	**Yes**	**Yes**	Use of continuous vasoactive drugs	**Yes**	**Yes**	**No**
Shock	**Yes**	**Yes**	**Yes**	Cardiopulmonary resuscitation	**Yes**	**Yes**	**Yes**
Oliguria non responsive to fluids or diuretics	**Yes**	**Yes**	**Yes**	Lactate >5mmL/L	**Yes**	**Yes**	**No**
Failure to form clots	**Yes**	**Yes**	**Yes**	pH<7.1	**Yes**	**Yes**	**No**
Loss of consciousness lasting more than 12 hours	**Yes**	**Yes**	**Yes**	**Respiratory dysfunction**			
Cardiac Arrest	**Yes**	**Yes**	**Yes**	Acute cyanosis	**Yes**	**Yes**	**Yes**
Stroke	**Yes**	**Yes**	**Yes**	Gasping	**Yes**	**Yes**	**Yes**
Uncontrollable fits / total paralysis	**Yes**	**Yes**	**Yes**	Respiratory rate >40 or <6/min	**Yes**	**Yes**	**Yes**
Jaundice in the presence of pre-eclampsia	**Yes**	**Yes**	**Yes**	Intubation/ventilation not related to anesthesia	**Yes**	**Yes**	**Yes**
Eclampsia	**No**	**Yes**	**Yes**	Oxygen saturation <90% for >60 min	**Yes**	**Yes**	**Yes**
Ruptured uterus	**No**	**Yes**	**Yes**	Pao_2_/FiO2 < 200 mm Hg	**Yes**	**Yes**	**No**
Sepsis or severe systemic infection	**No**	**No**	**Yes**	**Renal dysfunction**			
Pulmonary edema	**No**	**No**	**Yes**	Oliguria non responsive to fluids or diuretics	**Yes**	**Yes**	**Yes**
Severe complications of abortion	**No**	**No**	**Yes**	Dialysis for acute renal failure	**Yes**	**Yes**	**No**
Severe malaria	**No**	**No**	**Yes**	Creatinine ≥300μmol/L or ≥3.5 mg/dL	**Yes**	**Yes**	**Yes**
Severe pre-eclampsia with ICU admission	**No**	**No**	**Yes**	**Coagulation/hematological dysfunction**			
**Laboratory criteria**				Failure to form clots	**Yes**	**Yes**	**Yes**
Oxygen saturation <90% for >60 min	**Yes**	**Yes**	**Yes**	Transfusion of .. units of blood or red cells	**5**	**4**	**2**
Pao_2_/FiO2 < 200 mm Hg	**Yes**	**Yes**	**No**	Severe acute thrombocytopenia (<50.000/mL)	**Yes**	**Yes**	**Yes**
Creatinine ≥300μmol/L or ≥3.5 mg/dL	**Yes**	**Yes**	**Yes**	**Hepatic dysfunction**			
Bilirubin >100 μmol/L or >6.0 mg/dL	**Yes**	**Yes**	**No**	Jaundice in the presence of pre-eclampsia	**Yes**	**Yes**	**Yes**
pH<7.1	**Yes**	**Yes**	**No**	Bilirubin >100 μmol/L or >6.0 mg/dL	**Yes**	**Yes**	**No**
Lactate >5 mEq/mL	**Yes**	**Yes**	**No**	**Neurological dysfunction**			
Acute thrombocytopenia (<50 000 platelets/mL)	**Yes**	**Yes**	**Yes**	Loss of consciousness lasting more than 12 h	**Yes**	**Yes**	**Yes**
Loss of consciousness, glucose/ketoacids in urine	**Yes**	**Yes**	**Yes**	Loss of consciousness, glucose/ketoacids in urine	**Yes**	**Yes**	**Yes**
**Management-based criteria**				Stroke	**Yes**	**Yes**	**Yes**
Use of continuous vasoactive drugs	**Yes**	**Yes**	**No**	Uncontrollable fits / total paralysis	**Yes**	**Yes**	**Yes**
Hysterectomy following infection or hemorrhage	**Yes**	**Yes**	**Yes**	**Uterine dysfunction**			
Transfusion of … units of blood or red cells	**5**	**4**	**2**	Hysterectomy following infection or hemorrhage	**Yes**	**Yes**	**Yes**
Intubation and ventilation not related to anesthesia	**Yes**	**Yes**	**Yes**	**Additional parameters**			
Dialysis for acute renal failure	**Yes**	**Yes**	**No**	Eclampsia	**No**	**Yes**	**Yes**
Cardiopulmonary resuscitation	**Yes**	**Yes**	**Yes**	Ruptured uterus	**No**	**Yes**	**Yes**
Laparotomy other than CS	**No**	**No**	**Yes**	Sepsis or severe systemic infection	**No**	**No**	**Yes**
Laparotomy other than CS/ectopic pregnancy	**No**	**Yes**	**No**	Pulmonary edema	**No**	**No**	**Yes**
				Severe complications of abortion	**No**	**No**	**Yes**
				Severe malaria	**No**	**No**	**Yes**
				Severe pre-eclampsia with ICU admission	**No**	**No**	**Yes**
				Laparotomy other than CS	**No**	**No**	**Yes**
				Laparotomy other than CS/ectopic pregnancy	**No**	**Yes**	**No**

MNM – maternal near-miss, WHO – World Health Organization, SSA – sub-Saharan Africa, Yes - Criterion according to the specified tool, No - Not a criterion according to the specified tool

We used hospital births (86% of total births in Suriname) as a reference group. Data were collected through the childbirth books of all hospitals of babies with birth weight of at least 500 g.

### Outcome measures

The prevalence was calculated per 1000 live births and mortality-index was calculated by dividing maternal deaths (MD) with (MD+MNM). Causes were classified according to the International Classification of Diseases Maternal Mortality (ICD-MM) [19]. The underlying cause of maternal deaths and MNM diagnosis was the primary event in the chain-of-events [19,20]. Risk indicators were analyzed by comparing women who gave birth with MNM (numerator) to those who gave birth without MNM (denominator). No sample size calculation was performed due to the descriptive character of this study.

### Statistical analysis

SPSS version 25 (IBM, Armonk, NY, USA) was used and simple descriptive statistics were performed (frequencies, proportions, bar charts and pie charts). No data imputation was conducted as missing data was <5% and completely at random. Univariate binary logistic regression was performed to assess factors associated with MNM, reported in crude odds ratio (OR) with 95% confidence intervals (95% CI). Multivariate logistic regression included variables with *P* < 0.1 in the univariate analysis and the hypothesis-driven variables age, parity and ethnicity, and was reported in adjusted OR (aOR, 95% CI). Maternal near-miss was the dependent variable for the association with maternal characteristics. Each adverse perinatal outcome (preterm birth, low birth weight, low Apgar score and stillbirth) was the dependent variable for the associon with maternal-near miss. Possible explanatory factors such as BMI, socio-economic status and medical history could not be included due to the lack of this data in the reference group. The risk of MNM related to cesarean section (CS) could not be studied, due to bias by indication (CS could be both the cause and result of MNM).

### Ethical considerations

This research was approved by the ethical review board of the Surinamese Committee on Research Involving Human Subjects (#VG21-16) on October 4th, 2016. Informed consent was not deemed necessary as data were obtained from medical records without identification of the woman.

## RESULTS

### Prevalence, mortality-index and characteristics

**Figure 1:**
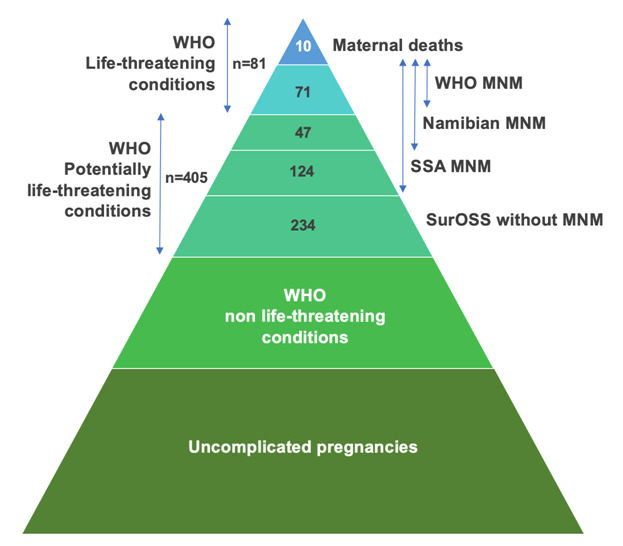
Number of women with maternal near miss according to the different tools.

During the one-year study period, there were 9114 live births and ten maternal deaths, which results in an MMR 110 per 100 000 live births. SurOSS identified 486 women with PLTC, of whom 234 had no MNM criteria (**Figure 1**). The primary health care centers reported ten women with PLTC who were not referred to a hospital, and none had MNM criteria. The WHO-tool identified 71 MNM (ratio 7.8 per 1000 LB, mortality-index 12% (n = 10/81)), the Namibian-tool identified 118 MNM (ratio 12.9 per 1000 LB, mortality-index 8% (n = 10/128)) and the SSA-tool 242 MNM (ratio 26.5 per 1000 LB, mortality-index 4% (n = 10/252)) (**Table 3**). Namibian and SSA-MNM identified all women with WHO-MNM. The three MNM-tools identified all maternal deaths. Patient characteristics are reported in Table 4. The proportion of women with MNM is highest in hospital I (34%-40% compared to 24% of total births), which is the only referral hospital. Women of Maroon-descent represent majority of MNM (37%-45%), while they account for 29% of total births.

**Table 3:** Demographics and maternal health indicators in Suriname

	Number		
**Deliveries**	9190		
**Total babies born**	9313		
**Live births**	9114		
**Maternal deaths**	10		
Maternal mortality ratio*	110		
**Near miss tools**	**WHO**	**Namibian**	**SSA**
**Maternal near miss, n =**	71	118	242
**MNM ratio†**	7.8	12.9	26.5
One MNM-criterion, n (%)	40 (56%)	79 (67%)	135 (56%)
Two or three MNM-criteria, n (%)	20 (28%)	26 (22%)	83 (34%)
Four or more MNM-criteria, n (%)	11 (16%)	13 (11%)	24 (10%)
Total amount of MNM-criteria	146	218	458
**Severe maternal outcomes, n**	81	128	252
**SMO ratio‡**	8.8	14.0	27.6
**Maternal near miss: mortality ratio**	7: 1	12: 1	24: 1
**Mortality index**§	12.3%	7.8%	4.0%
**Severity score,** mean (SD)‖	2.5 (2.2)	2.1 (2.0)	2.1 (1.8)

MNM – maternal near miss, WHO – World Health Organization, SSA – sub-Saharan Africa, SD – standard deviation

*Maternal mortality ratio: maternal deaths per 100 000 live births.

†Maternal near miss ratio: near miss cases per 1000 live births.

‡Severe maternal outcome ratio: near miss cases and maternal deaths per 1000 live births.

§Mortality index: number of maternal deaths divided by number of women with severe maternal outcomes (near miss and maternal deaths), expressed in percentages.

‖Average number of severity markers (near-miss criteria) in all SMO cases.

**Table 4:** Patient characteristics of women with MNM (not mutually exclusive) and all hospital births in the study period

	WHO		Namibian		SSA		Hospital births	
	n = 71	%	n = 118	%	n = 242	%	n = 9190	%
**Hospital:**								
I	24	33.8	47	39.8	81	33.5	2189	23.8
II	24	33.8	31	26.3	62	25.6	2647	28.8
III	15	21.1	24	20.3	58	24.0	2496	27.2
IV	7	9.9	12	10.2	29	12.0	1481	16.1
V	1	1.4	4	3.4	12	5.0	377	4.1
**Age (years):**								
<20	8	11.3	16	13.6	31	12.8	1214	13.2
20-35	43	60.6	79	66.9	163	67.4	6807	74.1
>35	20	28.2	23	19.5	48	19.8	995	10.8
**Parity:**								
Nullipara	22	31.0	46	39.0	83	34.3	3151	34.3
1-3	34	47.9	50	42.4	110	45.5	4785	52.1
≥4	15	21.1	22	18.6	49	20.2	1221	13.3
**Ethnicity:**							*Missing n = 43*	
Maroon	32	45.1	48	40.7	89	36.8	2639	28.9
Creole	14	19.7	27	22.9	56	23.1	1993	21.8
Hindustani	9	21.7	17	14.4	31	12.8	1737	19.0
Javanese	6	8.5	8	6.8	18	7.4	943	10.3
Mixed	7	9.9	10	8.5	27	11.2	1135	12.4
Indigenous	2	2.8	5	4.2	12	5.0	348	3.8
Other	1	1.4	3	2.5	9	3.7	352	3.8
**Residency:**	*Missing n = 3*		*Missing n = 7*		*Missing n = 17*			
Urban	57	83.8	96	86.5	194	86.2	-	-
Coastal	7	10.3	8	7.2	18	8.0	-	-
Rural	4	5.6	7	6.3	13	5.8	-	-
**Insurance:**	*Missing n = 1*		*Missing n = 3*		*Missing n = 5*			
State	49	70.0	79	68.7	167	70.5	-	-
Private	14	20.0	25	21.7	52	21.9	-	-
None	7	10.0	11	9.6	18	7.6	-	-
**Gestational age:**								
<22 weeks	9	12.7	13	11.0	25	10.2	-	-
22-28 weeks	3	4.2	5	4.2	16	6.6	160	1.7
28-36 weeks	30	42.3	52	44.1	89	36.8	1143	12.4
≥37 weeks	29	40.8	48	40.7	112	46.3	7887	85.8
**Pregnancy outcome:**								
Miscarriage	7	9.9	10	8.5	20	8.3	-	-
Ectopic	2	2.8	3	2.5	5	2.1	-	-
Vaginal delivery	34	47.9	53	44.9	119	49.2	6904	75.1
Instrumental delivery	1	1.4	2	1.7	2	0.8	123	1.3
Caesarean section	27	38.0	50	42.4	96	39.7	2163	23.5

### Differences between MNM criteria

**Figure 2:**
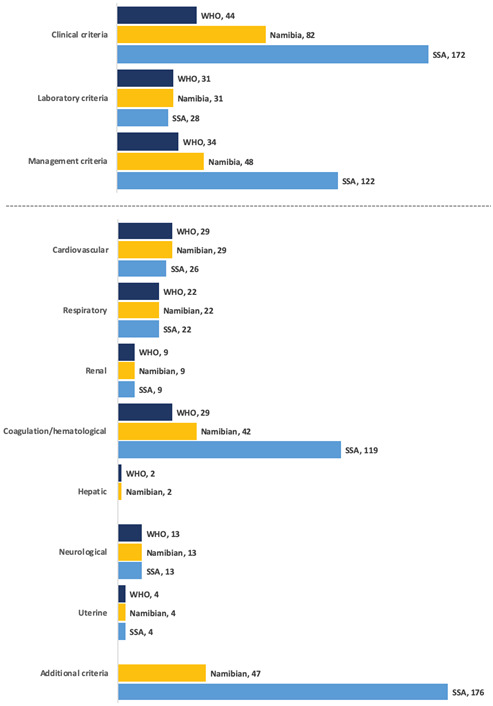
Number of women per maternal near miss category and tool, reported in events. *Coagulation dysfunction high for SSA-MNM and Namibian-MNM due to transfusion threshold of two units (n = 112) and four units (n = 31) respectively, instead of WHO-MNM threshold of five units of red blood cells (n = 15). †Additional criteria for Namibia-MNM included eclampsia (n = 44), uterine rupture (n = 1) and laparotomy other than for CS or ectopic pregnancy (n = 2). ‡Additional criteria for SSA-MNM included eclampsia (n = 44), uterine rupture (n = 1), severe sepsis (n = 40), pulmonary edema (n = 13), severe complications of abortion (n = 21), severe pre-eclampsia with ICU-admission (n = 103) and laparotomy other than CS (n = 6).

**Figure 2** (and Table S1 and S2 in the **Online Supplementary Document**) presents the distribution of MNM events. Laboratory MNM-events played a small role in the SSA-tool (9%, n = 28/322) compared to WHO-tool (28%, n = 31/109). The most important criteria were organ-dysfunction cardiovascular (27%), coagulation (27%) and respiratory (20%) for WHO-MNM, additional criteria (28%), coagulation (25%) and cardiovascular dysfunction (17%) for Namibian and additional criteria (48%) and coagulation dysfunction (32%) for the SSA tool.

**Figure 3:**
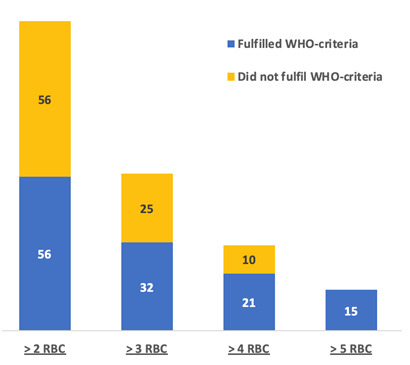
Number of women who received red blood cell (RBC) products and fulfilled WHO MNM-criteria.

Transfusion of >4 red blood cell (RBC) products (Namibian-criteria), instead of the WHO threshold >5, led to an additional 10 cases of women without any WHO-MNM, while transfusion of >2 RBC (SSA-criteria) led to an additional 91 women without any WHO-MNM being included (Figure 3). The transfusion of blood products was responsible for 21% (n = 15/71) of WHO-MNM, 26% (n = 31/118) of Namibian-MNM, and 46% (n = 112/242) of SSA-MNM. Eclampsia was not considered a WHO-MNM in 80% (n = 35/44) of cases as these women had no organ-dysfunction. Women with pre-eclampsia admitted to the ICU (n = 64) had no WHO-MNM criteria in 62% (n = 64/103).

**Box 1** illustrates disputable case examples of:

Box 1 **Case examples of women with and without a maternal near miss according to different criteria**

Severe morbidity according to SurOSS without any MNM criteria:

1. Woman admitted with HELLP syndrome at 30 weeks of gestation, delivered a girl of 950 grams by CS who died two days later.
2. Woman had a severe post-partum psychosis post-partum, walked away and was never seen again.
3. ICU admission for severe hypokalemia (1.8 mEq/L) and rhabdomyolysis (CK 10 000) due to pemba (clay) consumption.
4. Woman developed peri-partum cardiomyopathy three months post-partum and was admitted to ICU with moderate heart failure.

Namibian- and SSA-MNM, not included by the WHO-criteria:

1. A woman had three fits at home, was admitted with pre-eclampsia, stabilized and a caesarean section was performed. She had two fits post-partum.
2. A uterine rupture was discovered per-operatively in a woman with two previous CS. The woman received three packed cells and three fresh frozen plasma and was admitted to the ICU for severe hemorrhage (1500 mL). Her baby was in good condition.
3. Severe hemorrhage due to miscarriage at 19 weeks of gestation with hemoglobin level of 2.4 g/dL, for which patient was transfused 4 units RBC.
4. Laparotomy performed with suspicion for ectopic pregnancy, yet showed no ectopic mass. Post-operatively she developed a sepsis. Re-laparotomy showed an appendicitis and perforation of her intestines. An appendectomy and intestine repair were performed. Her pregnancy ended in a miscarriage.

Additional SSA-MNM, not included by the WHO- or Namibian-criteria:

1. Ruptured ectopic pregnancy, operated and complicated by a sepsis due to bilateral pneumonia for which she received intravenous antibiotics.
2. Severe pre-eclampsia, CS performed at 33 weeks. ICU admission for pulmonary edema (received 4-liter fluids in first 24 hour).
3. A woman from the interior with a septic and hemorrhagic miscarriage referred from interior clinic to hospital and arrived 12 hours later. She was admitted to the ICU, treated with intravenous antibiotics and was transfused 3 units of RBC.
4. Severe antepartum hemorrhage due to placental abruption at 36 weeks of gestation with vaginal birth of stillbirth baby. She was diagnosed with HELLP syndrome and transfused three units of RBC, six units of fresh frozen plasma and two platelet suspensions.

MNM and debatable severity:

1. Mild pre-eclampsia, uncomplicated term delivery with post-partum thrombocytopenia of 48 000 (platelets/mL) which resolved spontaneously. (included by all MNM-tools).
2. Transfusion of two units of RBC for post-partum hemorrhage of 700 mL and pre-delivery hemoglobin level of 9.4 g/dL (included by SSA MNM-tool).
3. Post-partum pre-eclampsia ICU-admission for monitoring of blood pressure and magnesium sulfate therapy. No complications (included by SSA MNM-tool).
4. In labor with fever, tachycardia with suspected chorioamnionitis for which antibiotics and CS. She recovered well (included by SSA MNM-tool).

1. women who were included in SurOSS but did not meet any MNM-criteria;
2. women included by Namibian- or SSA-MNM (not included by WHO-tool);
3. women included solely by SSA-MNM (not included by WHO or Namibian-tool); and
4. women with MNM in whom the severity of their disease is debatable.

### Underlying causes

**Figure 4:**
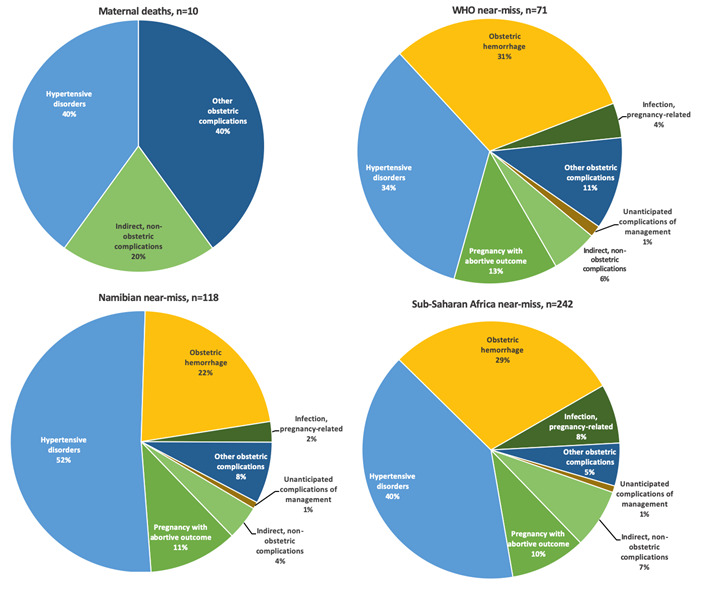
Primary underlying causes of maternal deaths and underlying diseases causing MNM according to the different tools. In the case of more than one near-miss event, the primary underlying cause was reported according to the ICD-MM guideline. *Maternal death “other obstetric complications” was caused by amniotic fluid embolism (n = 1), pulmonary embolism (n = 4) and peri-partum cardiomyopathy (n = 1).

Hypertensive disorders of pregnancy (HDP) was the most frequent primary diagnosis in women with MNM (34% WHO-MNM, 52% Namibian-MNM) (Figure 4). The case fatality-rate for HDP was 17% (n = 4/24, WHO-MNM), 7% (n = 4/61, Namibian-MNM), and 4% (n = 4/97, SSA-MNM). Women had multiple diagnosis in 8%-14%, for example: severe pre-eclampsia and thrombocytopenia followed by massive hemorrhage. The primary diagnosis of this case would be HDP (**Figure 4**). In Figure S1 and S2 in the **Online Supplementary Document** all diagnoses are reported (in number of events) and its underlying causes. The low number of maternal deaths (n = 10) limited analysis of case fatality rates for the other diseases. However, ‘other obstetric complications’ and ‘indirect, non-obstetric complications’ are responsible for 60% (n = 6/10) of maternal deaths, while they represent only 12%-17% of underlying causes of MNM (12% Namibian- and SSA-MNM and 17% WHO-MNM).

### Factors associated with MNM

For the WHO-criteria advanced maternal age and maroon ethnicity were associated with MNM, with respectively aOR = 2.59 (95%CI = 1.37-4.88) and aOR = 2.04 (95%CI = 1.15-3.61) after adjustment for age, parity, and ethnicity (**Table 5**). For the Namibian-criteria only maroon ethnicity was associated with MNM, aOR = 1.93 (95%CI = 1.25-2.99) after adjustment for age and parity (Table S3 in the **Online Supplementary Document**). For the SSA-criteria, next to advanced maternal age and maroon ethnicity, multiple pregnancy was significantly associated with MNM (aOR = 3.38, 95%CI = 1.68-6.81) (Table S4 in the **Online Supplementary Document**).

**Table 5:** Association between WHO maternal near-miss and maternal characteristics and perinatal outcomes (MNM n = 57, no MNM n = 9123)

	MNM*	No MNM	cOR	95% CI	*P*-value	aOR	95% CI	*P*-value
**Maternal characteristics**								
Teenage pregnancy	6/56 (10.7%)	1208/8950 (13.5%)	0.76	0.33-1.78	0.529	-		
Old maternal age >35 y	15/56 (26.8%)	978/8950 (10.9%)	3.12	1.79-5.44	<0.001	2.59	1.37-4.88	0.003
Maroon ethnicity	27/56 (48.2%)	2608/9082 (28.7%)	2.31	1.37-3.91	0.002	2.04	1.15-3.61	0.015
Nullipara	17/57 (29.8%)	3132/9091 (34.5%)	0.81	0.46-1.43	0.464	-		
Grande multipara (≥4)	16/57 (28.1%)	1203/9091 (13.2%)	2.56	1.43-4.57	0.002	1.63	0.83-3.21	0.158
Multiple pregnancy	1/57 (1.8%)	120/9123 (1.3%)	1.34	0.18-9.76	0.773	*-*		
**Perinatal outcomes**								
Low birth weight (<2500 g)	27/55 (49.1%)	1299/9076 (14.3%)	5.77	3.39-9.83	<0.001	1.02‡	0.41-2.57	0.960
Preterm birth (GA<37 w)	31/57 (54.4%)	1270/9123 (13.9%)	7.37	4.36-12.46	<0.001	2.65§	0.97-7.23	0.058
Low Apgar 5 min below 7	6/43 (14.0%)	227/8850 (2.6%)	6.16	2.57-14.74	<0.001	2.45‖	0.84-7.13	0.100
Late stillbirth (GA>28 w)	11/57 (19.3%)	111/9123 (1.2%)	19.42	9.80-38.47	<0.001	6.83‖	2.96-15.76	<0.001

GA – gestational age, MNM – maternal near miss, OR – odds ratio, CI – confidence interval, y – years, w – weeks, g – grams

*MNM is the dependent variable.

**†**MNM is the independent variable.

‡Adjusted for age, parity ethnicity, gestational age, Apgar score and stillbirth.

§Adjusted for age, parity ethnicity, birth weight, Apgar score and stillbirth.

‖Adjusted for age, parity, ethnicity, gestational age and birth weight.

The stillbirth rate among women with WHO-MNM is 193/1000 births (n = 11/57), and 153/1000 births (n = 15/98) and 110/1000 births (n = 23/209) for respectively Namibian-and SSA-MNM. Women without MNM had a stillbirth rate of 12/1000 births (n = 111/9123). Univariate analysis showed highly significant association between MNM and adverse perinatal outcomes (low birth weight, preterm birth, low Apgar score, and stillbirths) for the three MNM-criteria (**Table 5**, Table S3 and S4 in the Online Supplementary Document). In multivariate analysis only stillbirths remained significantly associated with MNM (WHO MNM: aOR = 6.83, 95%CI = 2.96-15.76, Namibian-MNM: aOR = 4.75, 95%CI = 2.34-9.62 and SSA-MNM: aOR = 3.98, 95%CI = 2.24-7.06) after adjustment for age, parity, ethnicity, gestational age and birth weight.

## DISCUSSION

This nationwide population-based study in Suriname demonstrated that for every woman who died, between seven and twenty-four women experienced MNM, depending on the type of MNM criteria used. The WHO-MNM criteria detected all maternal deaths and resulted in a mortality-index of 12% (n = 10/71), which justified the WHO terminology life-threatening. However, WHO-criteria underestimate the prevalence of severe complications as certain disease-based complications such as eclampsia with a high case fatality rate are not included. Namibian-MNM (which included disease- and intervention) criteria led to more cases and a lower mortality-index (8%, n = 10/118). Application of the SSA-MNM (excluded the majority of laboratory-criteria and added several disease-based criteria) resulted in more cases and a lower mortality-index (4%, n = 10/242). SSA-MNM may have overestimated the prevalence of MNM since not all complications directly threatened the woman's life. For all three MNM tools, hypertensive disorders of pregnancy contributed most frequently to MNM. Advanced maternal age and maroon ethnicity were associated with MNM and women with MNM had six times the odds of a stillbirth. The absence of applicable and globally comparable MNM-criteria prevents countries such as Suriname from the sustainable implementation of MNM-registration.

### Maternal near-miss criteria and obstetric transition stages

The fundamental aim of studying MNM is twofold: 1) to have globally comparable data on MNM and 2) to capture MNM cases and determine causes of MNM, which ultimately improve maternal health care and reduces maternal mortality [2]. The global universal WHO-MNM tool best achieves the first aim. Because MNM criteria are not as clear cut as other maternal health indicators (eg, MMR, stillbirth rate), underreporting is inevitable and will occur in all settings, most substantially in low-income settings [9-12,21,22]. If the purpose is to find solutions for the most critical problems associated with severe maternal outcomes (the second fundamental aim), local adaptations are unavoidable, though this subverts the first aim of globally comparable data.

Contextually-tailored MNM criteria may be the answer to achieve both fundamental aims of uniformity and applicability of MNM criteria. One contextual approach could be to incorporate the ‘obstetric transition framework’, which assimilates context-specific analysis and recommendations to improve the quality of care [23]. The framework, developed by Souza et al. (2014), describes the transition from higher MMR/fertility to low MMR/fertility within and between countries [23]. The problems and solutions for countries in obstetric transition stage I and II are incomparable to countries in stage III and IV. For example, in the first two stages, many maternal deaths occur and access to care and the availability of educated staff and resources play the most crucial role in reducing maternal mortality in these stages. Studying maternal mortality is of primary importance, and MNM studies play a limited role. However, if MNM studies are to be performed in these stages (eg, in rural settings with a low number of deaths), criteria should focus on 'direct' causes of maternal mortality (eg, severe hemorrhage and eclampsia). Stage III is known as a complicated stage as access to care is improved, and quality of care becomes a significant determinant of health outcomes. As maternal mortality decreases, MNM studies play an increasingly important role. The threshold of specific criteria (eg, blood transfusion) is higher than in stage I-II, and more focus is needed on 'indirect' causes. In stage IV, maternal mortality rates are low and severe outcomes are often the result of 'overmedicalization' and more high-risk pregnancies (high maternal age, non-communicable diseases, and pregnancies in women with severe comorbidities) [23]. MNM criteria in these stages need to focus on rare diseases with high case fatality rates (eg, abnormally invasive placentation, amniotic fluid embolism as proposed by the INOSS [18]), to reduce maternal mortality and reach the mostly aspirational stage V.

### Organ-based vs disease-based criteria

Case identification is more feasible when using disease-based criteria, than organ-dysfunction criteria (25-item list with many cut-off values) [2,17,24-26]. For example, clinicians easily identify a woman with eclampsia, while women with transient tachypnoea or thrombocytopenia are more difficult to identify. Another advantage of disease-based criteria is that the underlying problem is better understood and risk factors and case-fatality rates are easier to interpret. This makes it easier to identify gaps in the quality of care and find potential solutions to these problems. An illustrative example is the impact of disease-based criteria comparison between the Netherlands and the United Kingdom [24]. The observation that the Netherlands had a five-fold incidence of eclampsia, stemming from differences in clinical management, prompted rapid eclampsia incidence reductions through the implementation of different management protocols [25].

The WHO working group on Maternal Mortality and Morbidity Classification stated that organ dysfunction captures the severest diseases, and that disease-based criteria often have too low threshold to be considered ‘severe’ morbidity, and risk variation in definition and identification [6,7]. While organ-dysfunction are in the sequence of events leading from good health to death, it is not always measurable. An example is that only a small percentage of women with eclampsia in Suriname had measurable organ-dysfunction criteria, despite being very ill and nearly dead [26]. Although the inclusion threshold for near-miss is lower with most WHO disease-based criteria (eg, severe post-partum hemorrhage, sepsis and pre-eclampsia), it does not outweigh the benefits of clinical relevance and more feasible case identification. This would justify the initiation of a global consensus process for (higher threshold) definitions of severe morbidity and near-miss, as done by INOSS and the Core Outcomes in Women and Newborn (CROWN) Health initiative [18,27].

### Comparing prevalence and case-fatality rates

When comparing the prevalence of WHO-MNM to the region, Suriname has a similar prevalence to Brazilian referral hospitals (9.4 per 1000 live births) [28]. No comparison with other Latin America/Caribbean countries is possible, as the studies are conducted in single sites, have limited case numbers, and have modified the criteria [3,9,29]. The lack of comparison possibilities emphasizes how crucial it is to apply uniform MNM criteria (as proposed by the WHO-tool) to report the prevalence of MNM in countries reliably.

The proportion of maternal deaths and WHO-MNM due to hypertensive disorders of pregnancy in Suriname was high, 40%, and 34%, respectively. HDP are known to contribute significantly to maternal deaths in Latin America (22%), and for unclear reasons as the coverage of medication such as magnesium sulfate is adequate [4]. Currently, women with eclampsia are not included in MNM-criteria, while this disease is on the severest side of the spectrum of HDP. Only 80% of women with eclampsia in Suriname had WHO-MNM criteria, similar to previous studies [11,12]. Excluding eclampsia from MNM limits analysis of the factors contributing to the high burden of HDP. We are more likely to eliminate preventable maternal deaths if MNM studies were to include disease-based criteria with high case-fatality rates (such as eclampsia).

While MNM studies serve to monitor the quality of care by reporting numbers and trends, they barely facilitate the development of quality improvement strategies [3,8-12]. Near-miss audits are necessary to identify the lessons learned and to develop justified recommendations. The action and response to these findings and recommendations will finally reduce severe maternal (and perinatal) outcomes [25].

### Risk factors and adverse outcomes

Identifying risk factors is vital to guide interventions to reduce severe maternal and perinatal outcomes. However, as maternal near-miss consists of different diseases in different proportions, risk factors can be challenging to interpret. For example, while post-partum hemorrhage is associated with grand multiparity, eclampsia is prevalent among younger nulliparous women [29-31]. If the proportion is similar, the net result might be no association between parity and maternal near-miss (including both post-partum hemorrhage and eclampsia), as seen in our study. Similarly, old maternal age is a well-known risk factor for a broad spectrum of obstetric complications [31] and is strongly correlated with MNM in our study, as well as in a large multi-country study [32]. However, if the proportion of eclampsia-related MNM increases (in Namibian-MNM), the association between maternal age and MNM disappears. Equivalent to previous studies in Suriname, women of Maroon descent are at increased risk of adverse pregnancy outcomes as they have twice the odds of MNM compared to women of other ethnicities (for all three tools) [13,14]. These ethnic disparities may reflect socioeconomic inequalities and inequity within the health care system and need more attention. Ethnic disparities in severe maternal outcomes have also been reported in Brazil [33] and high-income countries (eg, the United States, the Netherlands, and Germany) [34-36].

Although it is clear that complications leading to MNM also contribute to adverse perinatal outcomes, the magnitude and causes of perinatal deaths among women with MNM are mainly unknown in low- and middle-income countries. The stillbirth rate among women with WHO-MNM in Suriname (193/1000 births) is higher than reported in Brazil (140/1000 births) [37] or other Latin American countries (128/1000 births) [30], and lower than in low-resource settings (eg, Ethiopia 284/1000 births [38]). The higher stillbirth rate among women with WHO-criteria (than Namibian- or SSA-criteria) further confirms that the WHO-tool comprises of the most clinically severe criteria. Improving national data collection of childbirth outcomes, disaggregated for maternal conditions, is necessary to improve identification and quantify factors that contribute to maternal complications and adverse perinatal outcomes.

Finally, compared to solely MNM registration, an audit of maternal near-miss is more likely to identify shortfalls in clinical practice and lead to improvements in both maternal and perinatal outcomes.

While MNM-tools register the number of severe maternal outcomes, an audit is necessary to reveal the actual ‘lessons to be learned’ [39]. Recommendations can be formed through these ‘lessons learned’, which encourage targeted action and response (eg, guideline updates, enabling policies and legislation, conduct research to fulfil knowledge gaps). This cycle of continuous evaluation, ‘maternal death and near-miss surveillance and response’, is essential in the elimination of preventable severe maternal outcomes and deserves a more prominent place in MNM studies [39-41].

### Strengths and limitations

Our study's strengths are the nationwide setting, prospective identification and robust data collection over a long period, and availability of background data on all deliveries. Several limitations need to be considered. First, we extracted data from patient records after discharge, and specific parameters (socioeconomic status, BMI) were unavailable. Second, reference data was limited to simple characteristics as no perinatal registry is yet in place and included no primary care and home births. Finally, we were not able to apply all SSA-criteria (eg, transfusion >2 RBC products) as SSA-criteria were published after the initiation of our study. The SSA-MNM prevalence is, therefore, higher than reported in our study.

## CONCLUSIONS

The MNM-ratio in middle-income country Suriname is 8 per 1000 live births according to the WHO-MNM tool. The Namibian- and SSA-MNM ratios are 13 and 27 per 1000 live birth. MNM may be underreported by the WHO (mortality-index 12%) and overreported by Namibian- and SSA-MNM (mortality-index 8% and 4%). Solutions to prevent under- and overreporting without compromising comparability can be to (1) create context-specific MNM-criteria per obstetric transition stage and; (2) use disease-based criteria. Advanced maternal age and maroon ethnicity were associated with MNM and women with MNM had six times the odds of a stillbirth. While MNM allows identification of women with severe outcomes, an audit is necessary to identify shortfalls in clinical practice and reduce severe maternal and perinatal outcomes.

## Additional material

Online Supplementary Document
