# Supplementary material for: Applicability of the WHO maternal near-miss tool: A nationwide surveillance study in Suriname
Source: J Glob Health. 2020 Oct 26;10(2):020429. doi: 10.7189/jogh.10.020429 (PMC7649043; doi:10.7189/jogh.10.020429)
Supplement: Online Supplementary Document [file jogh-10-020429-s001.pdf]

Table S1 MNM events by the WHO, Namibian and SSA clinical, laboratory and management criteria

	WHO<br>n=71	Namibian<br>n=118	SSA<br>n=242
**Clinical criteria**	**44**	**82**	**174**
Acute cyanosis	0	0	0
Gasping	2	2	2
Respiratory rate >40 or <6/min	11	11	11
Shock	24	24	24
Oliguria non responsive to fluids or diuretics	6	6	6
Failure to form clots	4	4	4
Loss of consciousness lasting more than 12 h	10	10	10
Cardiac Arrest	2	2	2
Stroke	2	2	2
Uncontrollable fits / total paralysis	6	6	6
Jaundice in the presence of pre-eclampsia	0	0	0
Eclampsia [a]	×	44	44
Ruptured uterus [a]	×	1	1
Sepsis or severe systemic infection [b]	×	×	40
Pulmonary edema [b]	×	×	13
Severe complications of abortion [b]	×	×	21
Severe malaria [b]	×	×	0
Severe pre-eclampsia with ICU admission [b]	×	×	103
**Laboratory criteria**	**31**	**31**	**28**
Oxygen saturation <90% for >60 minutes	8	8	8
PaO2/FiO2 <200 mmHg [c]	0	0	×
Creatinine ≥300μmol/l or ≥3.5 mg/dl	4	4	4
Bilirubin >100 μmol/l or > 6.0 mg/dl [c]	2	2	×
pH <7.1 [c]	2	2	×
Lactate >5 mEq/mL [c]	1	1	×
Acute thrombocytopenia (<50.000 platelets/ml)	17	17	17
Loss of consciousness and glucose/ketoacids in urine	1	1	1
**Management-based criteria**	**34**	**48**	**122**
Use of continuous vasoactive drugs [c]	7	7	×
Hysterectomy following infection or hemorrhage	4	4	4
Transfusion of .. units of blood or red cells	15[d]	31[e]	112[f]
Intubation and ventilation not related to anesthesia	15	15	15
Dialysis for acute renal failure	1	1	×
Cardiopulmonary resuscitation	2	2	2
Laparotomy other than for caesarian section [b]	×	×	6
Laparotomy other than for caesarean or ectopic pregnancy [g]	×	2	×

Legend
[a] Criterion added by Namibian and SSA-tools; [b] Criterion added by SSA-tool; [c] Criterion excluded by SSA-tool; [d] Five blood products; [e] Four blood products; [f] Two blood products; [g] Criterion added by Namibian-tool; × Not a criterion according to the specified tool

Table S2 MNM events by the WHO, Namibian and SSA organ-dysfunction criteria

	WHO n=71	Namibian n=118	SSA n=242
**Cardiovascular dysfunction**	**29**	**29**	**26**
Shock	24	24	24
Cardiac Arrest	2	2	2
Use of continuous vasoactive drugs [a]	7	7	×
Cardiopulmonary resuscitation	2	2	2
Lactate >5mml/l [a]	1	1	×
pH <7.1 [a]	2	2	×
**Respiratory dysfunction**	**22**	**22**	**22**
Acute cyanosis	0	0	0
Gasping	2	2	2
Respiratory rate >40 or <6/min	11	11	11
Intubation/ventilation not related to anesthesia	15	15	15
Oxygen saturation <90% for >60 minutes	8	8	8
PaO2/FiO2 <200 mmHg [a]	0	0	×
**Renal dysfunction**	**9**	**9**	**9**
Oliguria non responsive to fluids or diuretics	6	6	6
Dialysis for acute renal failure [a]	1	1	×
Creatinine ≥300µmol/l or ≥3.5 mg/dl	4	4	4
**Coagulation/hematological dysfunction**	**29**	**42**	**119**
Failure to form clots	4	4	4
Transfusion of .. units of blood or red cells	15[b]	31[c]	112[d]
Severe acute thrombocytopenia (<50.000/ml)	17	17	17
**Hepatic dysfunction**	**2**	**2**	**0**
Jaundice in the presence of pre-eclampsia	0	0	0
Bilirubin >100 µmol/l or > 6.0 mg/dl [a]	2	2	×
**Neurological dysfunction**	**13**	**13**	**13**
Loss of consciousness lasting more than 12 h	10	10	10
Loss of consciousness, glucose/ketoacids in urine	1	1	1
Stroke	2	2	2
Uncontrollable fits / total paralysis	6	6	6
**Uterine dysfunction**	**4**	**4**	**4**
Hysterectomy following infection or hemorrhage	4	4	4
**Additional parameters**	×	**47**	**176**
Eclampsia [e]	×	44	44
Ruptured uterus [e]	×	1	1
Sepsis or severe systemic infection [f]	×	×	40
Pulmonary edema [f]	×	×	13
Severe complications of abortion [f]	×	×	21
Severe malaria [f]	×	×	0
Severe pre-eclampsia with ICU admission [f]	×	×	103
Laparotomy other than for CS [f]	×	×	6
Laparotomy other than for CS or ectopic pregnancy [g]	×	2	×

Legend
[a] Criterion excluded by SSA-tool; [b] Five blood products; [c] Four blood products; [d] Two blood products; [e] Criterion added by Namibian and SSA-tools; [f] Criterion added by SSA-tool; [g] Criterion added by Namibian-tool; × Not a criterion according to the specified tool

Figure S1 Underlying causes of MNM for the different tools, number of events

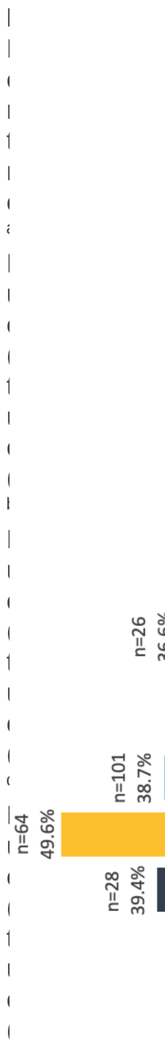

Figure S2 Distribution of the underlying causes and differences between the MNM criteria, number of events

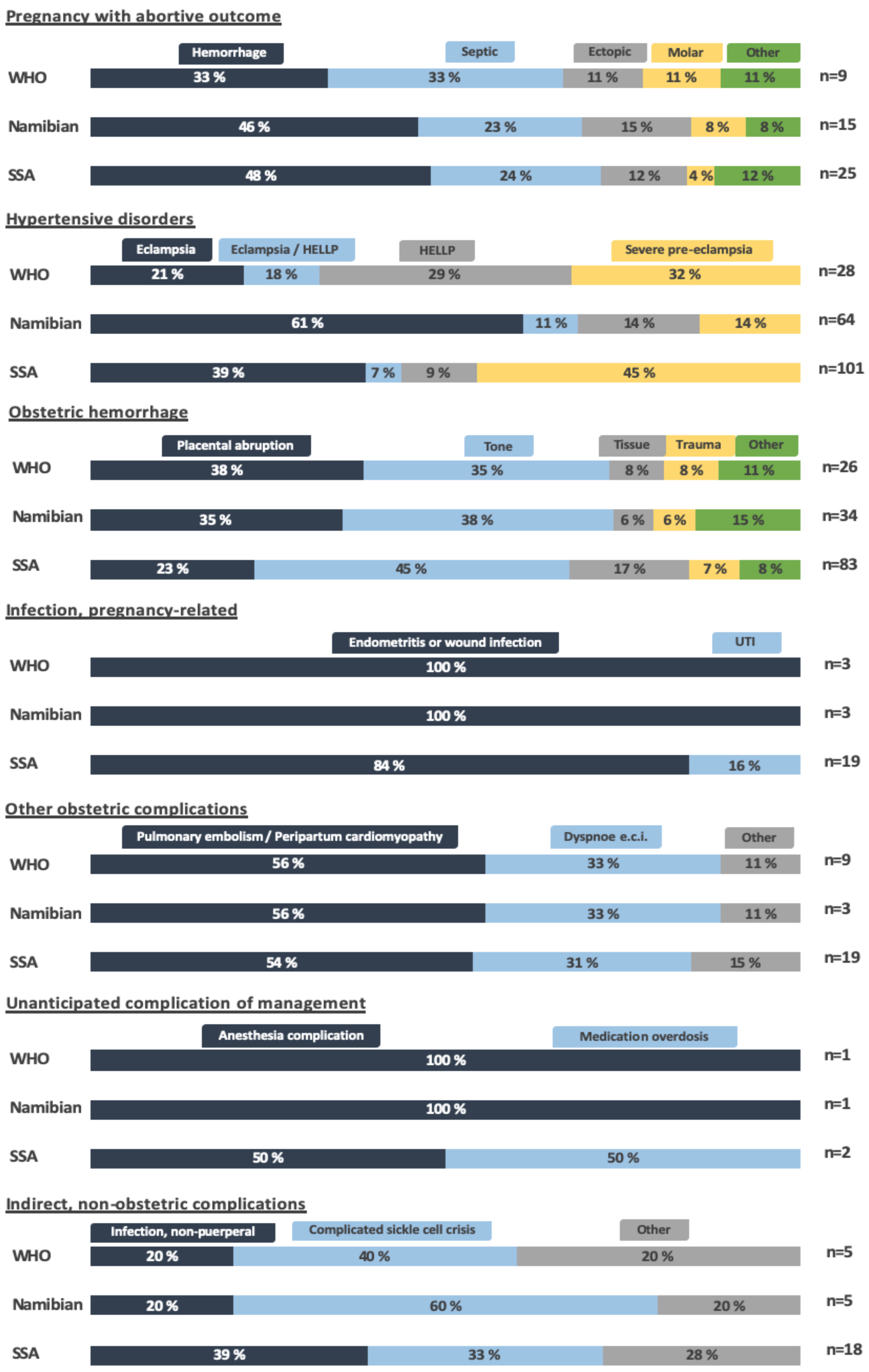

Table S3 Association between Namibian maternal near-miss and maternal characteristics and perinatal outcomes (MNM n=98, no MNM n=9082)

**Maternal characteristics**										
	**MNM[1]**	**No MNM**	**cOR**	**95% CI**		**p-value**	**aOR**	**95% CI**		**p-value**
Teenage pregnancy	13 / 98 (13.3%)	1201 / 8989 (13.4%)	0.99	0.55	1.78	0.978				
Old maternal age > 35yrs	20 / 97 (20.6%)	1275 / 8980 (14.2%)	1.57	0.96	2.58	0.075				
Maroon ethnicity	43 / 97 (44.3%)	2592 / 9041 (28.7%)	1.98	1.32	2.97	**0.001**	1.93	1.25	2.99	**0.003**
Nullipara	39 / 98 (39.8%)	3110 / 9050 (34.4%)	1.26	0.84	1.90	0.261				

Grande multipara (≥ 4)	22 / 98 (22.4%)	1197 / 9050 (13.2%)	1.90	1.18	3.06	**0.009**	1.47	0.86	2.53	0.162
Multiple pregnancy	1 / 98 (1.0%)	120 / 9082 (1.3%)	0.77	0.11	5.57	0.796				
**Perinatal outcomes**										
	**MNM[2]**	**No MNM**	**cOR**	**95% CI**		**p-value**	**aOR**	**95% CI**		**p-value**
Low birth weight (<2500 g)	48 / 94 (51.1%)	1278 / 9037 (14.1%)	6.34	4.21	9.53	**<0.001**	1.34[3]	0.67	2.67	0.41
Preterm birth (GA <37w)	53 / 98 (54.1%)	1248 / 9082 (13.7%)	7.39	4.95	11.05	**<0.001**	**2.25[4]**	**1.11**	**4.56**	**0.025**
Low Apgar 5 min below 7	9 / 78 (11.5%)	224 / 8815 (2.5%)	5.00	2.47	10.15	**<0.001**	1.91[5]	0.83	4.39	0.128
Late stillbirth (GA>28w)	15 / 98 (15.3%)	107 / 9082 (1.2%)	15.2	8.5	27.1	**<0.001**	**4.74[5]**	**2.34**	**9.62**	**<0.001**

Legend
[1] MNM is the dependent variable
[2] MNM is the independent variable
[3] Adjusted for age, parity ethnicity, gestational age, Apgar score and stillbirth
[4] Adjusted for age, parity ethnicity, birth weight, Apgar score and stillbirth
[5] Adjusted for age, parity, ethnicity, gestational age and birth weight
GA: gestational age

Table S4 Association between Sub-Sahara African maternal near-miss and maternal characteristics and perinatal outcomes (MNM n=209, no MNM n=8971)

**Maternal characteristics**										
	**MNM[1]**	**No MNM**	**cOR**	**95% CI**		**p-value**	**aOR**	**95% CI**		**p-value**
Teenage pregnancy	23 / 208 (11.1%)	1191 / 8879 (13.4%)	0.80	0.52	1.24	0.324				
Old maternal age > 35yrs	45 / 207 (21.7%)	1250 / 8870 (14.1%)	**1.69**	**1.21**	**2.37**	**0.002**	**1.50**	**1.03**	**2.20**	**0.036**
Maroon ethnicity	76 / 208 (36.5%)	2559 / 8930 (28.7%)	**1.43**	**1.08**	**1.91**	**0.014**	**1.42**	**1.04**	**1.94**	**0.027**
Nullipara	74 / 208 (35.6%)	2075 / 8940 (34.4%)	1.05	0.79	1.40	0.723				
Grande multipara (≥ 4)	43 / 208 (20.7%)	1176 / 8940 (13.2%)	**1.72**	**1.22**	**2.42**	**0.002**	1.36	0.93	1.99	0.113
Multiple pregnancy	9 / 209 (4.3%)	112 / 8971 (1.2%)	**3.56**	**1.78**	**7.12**	**<0.001**	**3.38**	**1.68**	**6.81**	**0.001**
**Perinatal outcomes**										
	**MNM[2]**	**No MNM**	**cOR**	**95% CI**		**p-value**	**aOR**	**95% CI**		**p-value**
Low birth weight (<2500 g)	92 / 202 (45.5%)	1234 / 8929 (13.8%)	**5.22**	**3.93**	**6.92**	**<0.001**	1.45[3]	0.92	2.92	0.114
Preterm birth (GA <37w)	101 / 209 (48.3%)	1200 / 8971 (13.4%)	**6.06**	**4.59**	**8.00**	**<0.001**	**2.57[4]**	**1.62**	**4.11**	**<0.001**
Low Apgar 5 min below 7	20 / 172 (11.6%)	213 / 8721 (2.4%)	**5.26**	**3.23**	**8.54**	**<0.001**	**2.41[5]**	**1.36**	**4.30**	**0.003**
Late stillbirth (GA>28w)	23 / 209 (11.0%)	99 / 8971 (1.1%)	**11.08**	**6.88**	**17.84**	**<0.001**	**3.98[5]**	**2.24**	**7.06**	**<0.001**

Legend
[1] MNM is the dependent variable
[2] MNM is the independent variable
[3] Adjusted for age, parity ethnicity, gestational age, Apgar score and stillbirth
[4] Adjusted for age, parity ethnicity, birth weight, Apgar score and stillbirth
[5] Adjusted for age, parity, ethnicity, gestational age and birth weight
GA: gestational age
